# Factors associated with non-essential workplace attendance during the COVID-19 pandemic in the UK in early 2021: evidence from cross-sectional surveys

**DOI:** 10.1016/j.puhe.2021.07.002

**Published:** 2021-09

**Authors:** S. Michie, H.W.W. Potts, R. West, R. Amlȏt, L.E. Smith, N.T. Fear, G.J. Rubin

**Affiliations:** a University College London, Centre for Behaviour Change, United Kingdom; b University College London, Institute of Health Informatics, United Kingdom; c University College London, Department of Behavioural Science and Health, United Kingdom; d King's College London, Department of Psychological Medicine, United Kingdom; e Public Health England, Behavioural Science Team, Emergency Response Department Science and Technology, United Kingdom; f NIHR Health Protection Research Unit in Emergency Preparedness and Response, United Kingdom; g King's College London, King's Centre for Military Health Research and Academic Department of Military Mental Health, United Kingdom; h Porton Down, Salisbury, Wiltshire, United Kingdom

**Keywords:** COVID-19, Workplace attendance, Sociodemographic variables, Personal circumstances

## Abstract

**Objectives:**

Working from home where possible is important in reducing the spread of COVID-19. In early 2021, a quarter of people in England who believed they could work entirely from home reported attending their workplace. To inform interventions to reduce this, this study examined associated factors.

**Study design:**

Data from the ongoing COVID-19 Rapid Survey of Adherence to Interventions and Responses survey series of nationally representative samples of people in the UK aged 16+ years in January–February 2021 were used.

**Methods:**

The study sample was 1422 respondents who reported that they could work completely from home. The outcome measure was self-reported workplace attendance at least once during the preceding week. Factors of interest were analysed in three blocks: 1) sociodemographic variables, 2) variables relating to respondents’ circumstances and 3) psychological variables.

**Results:**

26.8% (95% confidence interval [CI] = 24.5%–29.1%) of respondents reported having attended their workplace at least once in the preceding week. Sociodemographic variables and living circumstances significantly independently predicted non-essential workplace attendance: male gender (odds ratio [OR] = 1.85, 95% CI = 1.33–2.58); dependent children in the household (OR = 1.65, 95% CI = 1.17–2.32); financial hardship (OR = 1.14, 95% CI = 1.08–1.21); lower socio-economic grade (C2DE; OR = 1.65, 95% CI = 1.19–2.53); working in sectors such as health or social care (OR = 4.18, 95% CI = 2.56–6.81), education and childcare (OR = 2.45, 95% CI = 1.45–4.14) and key public service (OR = 3.78, 95% CI = 1.83–7.81) and having been vaccinated (OR = 2.08, 95% CI = 1.33–3.24).

**Conclusions:**

Non-essential workplace attendance in the UK in early 2021 during the COVID-19 pandemic was significantly independently associated with a range of sociodemographic variables and personal circumstances. Having been vaccinated, financial hardship, socio-economic grade C2DE, having a dependent child at home and working in certain key sectors were associated with higher likelihood of workplace attendance.

## Introduction

Workplaces have been identified as settings for the spread of COVID-19,^1^^,^^2^ with outbreaks and clusters being reported in a variety of occupational settings in the UK and Europe.^3^ Factors associated with workplace outbreaks have been found to include occupations associated with low socio-economic status, workers in essential settings who cannot work from home and workplaces without robust ‘COVID-19–safe’ policies and procedures.^1^ Understanding the factors that contribute to people attending the workplace when they do not need to will inform interventions to reduce this practice.

In the UK, national lockdown restrictions were interspersed with regional tiered restrictions to reduce the nature and extent of interpersonal contacts that lead to infectious disease transmission. These restrictions have taken various forms and have had varied effects, with stringent restrictions shown to outperform more relaxed restrictions in terms of their impact on behaviour, hospitalisations and deaths.^4^ It is therefore likely that contact between people in workplaces contributes to transmission within workplaces and between workplaces and homes.^5^ Indeed, 40% of people testing positive for COVID-19 reported prior workplace or education activity, and the emergence of clusters has been interpreted to be the result of widespread failure to control risks of airborne and surface transmission in workplaces.^6^^,^^7^

In many cases, attending the workplace is not essential, either because workers can be furloughed or because people can work from home. However, in the lockdown in early 2021, the third lockdown in England, the Office for National Statistics reported that 48% of working-age adults had travelled to work at least once in the past seven days,^8^ compared with 37% in the first lockdown in May 2020.^9^ This may have been associated with more furlough requests having been turned down.^10^

Concern has been raised that many people who are attending work at present do not need to do so and could instead work from home.^11^ The UK COVID-19 Rapid Survey of Adherence to Interventions and Responses [CORSAIR] national survey of 2000 people found that in February 2021, 35% of those who could work from home had been out to work at least once in the previous week, with 12% at least five times.^12^ A national poll of nearly 1000 employees commissioned by the Trades Union Congress (TUC) and conducted by YouGov found that 19% of those still working were going into offices or other workplaces for part or all of their working week despite being able to work from home.^13^ The main reason given was pressure from employers (40%).

There may be many factors influencing workplace attendance when home working is possible. These may include factors relating to sociodemographic characteristics such as age, gender and ethnic group. For example, young people may perceive themselves to be less at risk from COVID-19 and therefore are more likely to attend their workplace. Second, they may include factors relating to people's circumstances, for example, the type of job that they do or their living circumstances. For example, people may feel pressure from their employer to attend the workplace or worried about losing their job if they work from home, or they may be able to work from home but not have adequate equipment to make this easy. And third, they may include factors relating to knowledge and attitudes, for example, being less concerned about the harmfulness of COVID-19 after vaccination.

Uptake of vaccination has been high in the UK, unlike in some other countries.^14^ The rollout of the vaccination programme in the UK has raised concerns that it may create a sense of reassurance about getting, being harmed by and spreading COVID-19 and that this may lead to more risky behaviours.^15^ This concern arose from evidence of risk compensation and reduced protective behaviours after vaccination from other programmes,^16^^,^^17^ in addition to the UK survey finding that 29% of respondents said that they would adhere less strictly than before vaccination^18^ and 22% said they believed that those who had been vaccinated should not be subject to any more coronavirus restrictions.^19^ Real-world data have shown spikes in infection rates in the nine days after vaccination in both Israel^20^ and the UK,^21^ with some suggesting that this may reflect more risky behaviours after vaccination.^22^

Understanding factors influencing non-essential workplace attendance during a critical period in the COVID-19 pandemic in the UK could provide useful information to inform interventions aimed at reducing it. This study aimed to examine factors associated with attending the workplace amongst those who could work entirely from home. To do this, we analysed data from the CORSAIR study, designed to collect information during the pandemic to help inform policies and interventions.^23^ This is an ongoing series of surveys carried out weekly or fortnightly with nationally representative samples of UK-based adults. Questions are added in specific waves to address issues of concern at that time.

The variables of interest were analysed according to a model whereby the sociodemographic factors may be expected to have their effect through, and be supplemented by, situational factors which may, in turn, have their effect through and be supplemented by psychological factors. In practice, because it is not possible to measure all the potential predictors of unnecessary workplace attendance with sufficient accuracy, the more distal factors may independently predict attendance, operating through more proximal factors that have not been measured or have not been measured with sufficient precision.

The research question addressed by this study was which variables independently predict non-essential workplace attendance in terms of 1) sociodemographic factors only, 2) sociodemographic factors, personal circumstances and situational factors and 3) sociodemographic factors, personal circumstances and psychological factors?

## Methods

### Design

The study used data from an ongoing series of cross-sectional online surveys, conducted by BMG Research, a Market Research Society Company Partner, on behalf of the Department of Health and Social Care. The survey began in January 2020 and has continued into 2021 either weekly or fortnightly. Further details are described in the study by Smith et al.^23^ Three waves of the survey were used in which a question about working from home was included: 25–26 January 2021, 8–9 February 2021 and 22–23 February 2021 (waves 42, 43 and 44). Because of the need for rapid turnaround for data collection during a rapidly evolving crisis, the surveys used standard opinion polling methods including non-probability sampling, an approach common within market research, political polling and social science. Quota samples aim to minimise bias by filling predetermined targets so that the social and demographic characteristics of the participants match the national population. As such, participants who belong to a quota that has already been met are prevented from completing the survey. Therefore, response rate is not a useful indicator of response bias in quota samples.

### Setting

United Kingdom.

### Participants

The sample included those who said they could fully work at home, recruited from two specialist online panel providers, Respondi and Savanta.^23^ Participants were eligible for the study if they were aged 16 years or older and lived in the UK. If a respondent completed the survey, they were unable to participate in the following three waves. Quotas were applied based on age and gender (combined) and reflected targets based on data from the Office for National Statistics.^24^ Therefore, the sociodemographic characteristics of participants in each survey wave were broadly similar to those in the UK general population. Participants were reimbursed in points which could be redeemed in cash, gift vouchers or charitable donations (up to 70p per survey). The total sample from the three survey waves was 6033, of whom 3271 reported that they were in work. Of these, 1422 reported that they could fully work at home, and this formed the sample for the present study.

### Measures

For the outcome measure, participants were asked to ‘Please enter the number of times you have been out of your home in the last seven days, for each of the following reasons’ with ‘to go out to work’ listed as one of the reasons. Responses were dichotomised into any workplace attendance (1) versus none (0). Because the sample had reported that they could work fully at home, we have taken this as a measure of non-essential workplace attendance.

Potential predictor variables were 1) sociodemographic variables (gender, age, educational level, ethnic group,^25^ English not as first language, Government Office Region in England plus Scotland, Wales and Northern Ireland, survey wave), 2) variables relating to the respondents’ circumstances (marital status, living alone, having a dependent child in the household, employment status, manual occupation of main earner [socio-economic grade C2DE^26^], working in one of a number of potentially risky types of workplace, Index of Multiple Deprivation in quartiles,^27^ COVID-19–related financial hardship, having a chronic illness that heightens risk of severe illness from COVID-19, having a household member who has a chronic illness, having been vaccinated) and 3) psychological variables (worry about COVID-19, perceived risk to self from COVID-19, believing that one has had COVID-19, belief that government information on COVID-19 is biased and willingness to leave home if they had symptoms). Full details of all the measures are provided in the Supplementary file.

The categories used in the question on work sector were chosen to identify those who work in a key occupational sector. People were asked to ‘indicate if you work in any of the following sectors or roles? Please include any voluntary work’. This categorisation may thus not represent a person's main employment.

### Ethics

This work was conducted as part of service evaluation of the marketing and communications run by the Department of Health and Social Care and so did not require ethical approval.

### Patient and public involvement

Lay members served on the advisory group for the project that developed our prototype survey material; this included three rounds of qualitative testing. Owing to the rapid nature of this research, the public was not involved in the further development of the materials during the COVID-19 pandemic.

### Power

The sample size of approximately 1400 (depending on the analysis) provided >90% power to detect an odds ratio (OR) representing a ‘small’ effect size (f^2^ = 0.02) with 2-tailed alpha of 0.05 in a multivariable regression with 24 potential predictor variables entered together.

### Analysis

The sample was weighted by age, gender and Government Office Region to match the UK population aged 16+ years. The full weighted sample size was 1428. Missing values were excluded on an analysis-by-analysis basis leading to smaller sample sizes in some cases. Frequencies and percentages were calculated for prevalence, and multivariable logistic regressions were undertaken to determine associations between the primary outcome and predictor variables.

First, all predictor variables in Block 1 (sociodemographic variables) were entered. Then, variables from Block 2 (respondents’ circumstances) were added to the model. Then, variables from Block 3 (psychological variables) were added.

The analyses plan was not preregistered, and so the analyses should be considered exploratory.

## Results

Of a weighted sample of 1428, 26.7% (n = 382, 95% confidence interval [CI] = 24.5–29.1) attended the workplace in the preceding seven days. Table 1 shows the characteristics of the sample, overall and by attendance at their workplace.

**Table 1:** Participant characteristics and comparison of those attending and not attending their workplace.

Variable	Attended workplace^a^ % (n)	Did not attend workplace^a^ % (n)	All^b^^,^^c^ % (n)
**Block 1: sociodemographic variables**			
Wave, % (n)			
Wave 1 (25–26 January 2021)	25.7 (132)	74.3 (382)	36.0 (514)
Wave 2 (8–9 February 2021)	25.1 (114)	74.9 (341)	31.9 (455)
Wave 3 (22–23 February 2021)	29.5 (135)	70.5 (323)	32.1 (458)
Region, % (n)			
East Midlands	25.8 (24)	74.2 (69)	6.5 (93)
East of England	22.1 (30)	77.9 (106)	9.5 (136)
London	37.2 (108)	62.8 (182)	20.3 (290)
North East	29.4 (15)	70.6 (36)	3.6 (51)
North West	28.4 (42)	71.6 (106)	10.4 (148)
Northern Ireland	26.5 (9)	73.5 (25)	2.4 (34)
Scotland	20.6 (27)	79.4 (104)	9.2 (131)
South East	15.5 (26)	84.5 (142)	11.8 (168)
South West	21.7 (20)	78.3 (72)	6.4 (92)
Wales	26.8 (15)	73.2 (41)	3.9 (56)
West Midlands	28.8 (38)	71.2 (94)	9.2 (132)
Yorks & Humber	27.6 (27)	72.4 (71)	6.9 (98)
Gender, % (n)^d^			
Female	19.8 (125)	80.2 (505)	44.2 (630)
Male	32.2 (256)	67.8 (538)	55.8 (794)
Age, mean (SD)^d^	36.5 (11.6)	42.1 (13.1)	40.6 (13.0)
Educational level, % (n)			
Non-degree level	26.5 (177)	73.5 (492)	46.8 (669)
Degree level	27.0 (205)	73.0 (554)	53.2 (759)
Ethnic group, % (n)^d^			
White British	24.0 (271)	76.0 (856)	79.2 (1127)
White ethnic minorities	35.2 (37)	64.8 (68)	7.4 (105)
Ethnic minorities (excluding White minorities)	38.2 (73)	61.8 (118)	13.4 (191)
Language, % (n)			
English as 1st language	25.8 (327)	74.2 (941)	88.7 (1268)
English not as 1st language	34.2 (55)	65.8 (106)	11.3 (161)
**Block 2: Personal circumstances**			
Marital status, % (n)			
Not married/partnered	24.9 (120)	75.1 (362)	33.9 (482)
Married/partnered	27.4 (258)	72.6 (682)	66.1 (940)
Living social status, % (n)			
Live with other(s)	27.3 (327)	72.8 (873)	84.0 (1200)
Live alone	24.0 (55)	76.0 (174)	16.0 (229)
Children at home, % (n)^d^			
No dependent children	18.2 (140)	81.8 (628)	53.8 (768)
Dependent children	36.7 (242)	63.3 (418)	46.2 (660)
Employment status, % (n)^d^			
Full-time	29.0 (339)	71.0 (828)	81.7 (1167)
Part-time	15.5 (24)	84.5 (131)	10.9 (155)
Self-employed	17.9 (19)	82.1 (87)	7.4 (106)
Socio-economic grade, % (n)^d^			
Non-manual occupation	21.7 (234)	78.3 (846)	76.5 (1080)
Manual occupation	43.4 (144)	56.6 (188)	23.5 (332)
Work sector, % (n)^d^			
Other	15.4 (103)	84.6 (564)	46.7 (667)
Health & social care	53.0 (105)	47.0 (93)	13.9 (198)
Education & childcare	33.6 (45)	66.4 (89)	9.4 (134)
Key public service	43.3 (26)	56.7 (34)	4.2 (60)
Local & national govt	18.6 (18)	81.4 (79)	6.8 (97)
Food & essential goods	37.0 (20)	63.0 (34)	3.8 (54)
Public safety & security	44.0 (11)	56.0 (14)	1.7 (25)
Transport	38.1 (16)	61.9 (26)	2.9 (42)
Utilities, comms & finance	25.0 (38)	75.0 (114)	10.6 (152)
Index of multiple deprivation, % (n)			
1st quartile (lowest)	21.1 (62)	78.9 (232)	20.6 (294)
2nd quartile	23.1 (83)	76.9 (277)	25.2 (360)
3rd quartile	24.2 (93)	75.8 (291)	26.9 (384)
4th quartile (highest)	37.1 (145)	62.9 (246)	27.4 (391)
Financial hardship, mean (SD, n)	9.50 (3.01, 371)	7.75 (2.92, 1016)	8.21 (3.05, 1387)
Chronic illness of self, % (n)			
No chronic illness	25.5 (306)	74.5 (894)	85.5 (1200)
Chronic illness	34.5 (70)	65.5 (133)	14.5 (203)
Chronic illness of household member, % (n)			
No chronic illness	26.8 (330)	73.2 (903)	87.9 (1233)
Chronic illness	27.1 (46)	72.9 (124)	12.1 (170)
Vaccination status, % (n)^d^			
Not been vaccinated	23.1 (277)	76.9 (921)	84.0 (1198)
Been vaccinated	45.4 (104)	54.6 (125)	16.0 (229)
**Block 3: Psychological variables**			
Worried about COVID-19, mean (SD, n)	2.35 (1.14, 382)	2.46 (1.10, 1046)	2.43 (1.11, 1428)
Risk of COVID-19 to self, mean (SD, n)	3.23 (1.20, 380)	3.15 (1.10, 1034)	3.19 (1.13, 1414)
Believe Gov info is biased, mean (SD, n)^d^	2.62 (1.14, 382)	2.92 (1.23, 1046)	2.84 (1.21, 1428)
Believe had COVID-19, % (n)^d^			
Not had COVID-19 or do not know	24.0 (264)	76.0 (836)	77.0 (1100)
Had COVID-19	36.0 (118)	64.0 (210)	23.0 (328)
Willing to leave home if with symptoms, % (n)^d^			
Not willing	19.1 (160)	80.9 (677)	65.2 (837)
Willing	30.4 (136)	69.6 (311)	34.8 (447)

SD, standard deviation.

a Percentages sum to 100 across ‘Attended workplace’ and ‘Did not attend workplace’.

b Percentages sum to 100 down level of predictor variable.

c Sample sizes vary because of missing values and rounding of weighted totals.

d Workplace attendance differs across values of predictor variable *P* < 0.001 by χ^2^ test for percentages or analysis of variance for means.

Table 2 shows the results of the multivariable logistic regression analysis in the three blocks. In the first block, age, gender, ethnic group and educational level were predictive of workplace attendance. With the addition of the second block, age and educational level were no longer significant predictors of workplace attendance, while having dependent children at home, socio-economic grade C2DE, financial hardship, having been vaccinated and working in a certain sectors (health and social care, education and childcare, key public services, food and essential goods, public safety and security and transport) were associated with higher likelihood of workplace attendance. Working part-time or being self-employed was associated with lower likelihood of workplace attendance. None of the psychological variables included were significantly associated with workplace attendance.

**Table 2:** Factors associated with attending the workplace at least once in the previous 7 days using multivariable logistic regression.^a^

Predictor	Block 1 (odds ratio, 95% CI)	Block 2 (odds ratio, 95% CI)	Block 3 (odds ratio, 95% CI)
**Block 1: sociodemographic variables**			
Wave			
Wave 1 (Ref)			
Wave 2	0.95, 0.66–1.35	0.89, 0.61–1.31	0.90, 0.61–1.33
Wave 3	1.35, 0.97–1.88	1.13, 0.78–1.63	1.18, 0.81–1.71
Region			
East Midlands (Ref)			
East of England	0.69, 0.33–1.45	0.62, 0.28–1.41	0.61, 0.27–1.39
London	1.10, 0.58–2.09	0.96, 0.48–1.94	0.95, 0.47–1.93
North East	1.25, 0.53–2.95	1.24, 0.49–3.18	1.28, 0.50–3.29
North West	1.14, 0.57–2.28	1.04, 0.49–2.23	1.02, 0.47–2.22
Northern Ireland	1.37, 0.51–3.69	1.09, 0.37–3.25	1.01, 0.33–3.05
Scotland	0.71, 0.34–1.47	0.72, 0.32–1.58	0.71, 0.32–1.57
South East	0.58, 0.29–1.19	0.56, 0.25–1.23	0.55, 0.25–1.22
South West	0.95, 0.44–2.02	0.73, 0.31–1.71	0.74, 0.31–1.75
Wales	1.22, 0.52–2.86	1.29, 0.51–3.27	1.32, 0.52–3.35
West Midlands	0.96, 0.48–1.93	0.77, 0.36–1.67	0.74, 0.34–1.61
Yorks & Humber	1.02, 0.47–2.19	0.96, 0.41–2.22	0.93, 0.40–2.19
Gender			
Male (Ref)			
Female	**1.89, 1.42**–**2.53**	**1.83, 1.32**–**2.54**	**1.85, 1.33**–**2.58**
Age^b^	**0.97, 0.96**–**0.99**	0.99, 0.98–1.01	0.99, 0.98–1.01
Educational level			
Non-degree level (Ref)			
Degree level	**0.70, 0.52**–**0.94**	0.78, 0.56–1.09	0.76, 0.54–1.07
Ethnic group			
White British (Ref)			
White ethnic minorities	1.75, 0.91–3.35	**2.36, 1.15**–**4.83**	**2.39, 1.16**–**4.91**
Ethnic minorities (excluding White minorities)	**1.68, 1.09**–**2.60**	1.49, 0.92–2.41	1.49, 0.92–2.42
Language			
English as 1st language (Ref)			
English not as 1st language	0.78, 0.45–1.37	0.66, 0.36–1.24	0.69, 0.37–1.29
**Block 2: Personal circumstances**			
Marital status	–		
Not married/partnered (Ref)			
Married/partnered		1.20, 0.79–1.83	1.20, 0.79–1.83
Living social status	–		
Live with other(s) (Ref)			
Live alone		0.83, 0.48–1.43	0.82, 0.47–1.43
Dependent children	–		
No dependent children (Ref)			
Dependent children		**1.60, 1.14**–**2.24**	**1.65, 1.17**–**2.32**
Employment status	–		
Full-time (ref)			
Part-time		**0.40, 0.21**–**0.73**	**0.38, 0.20**–**0.70**
Self-employed		**0.43, 0.21**–**0.87**	**0.43, 0.21**–**0.89**
Socio-economic grade			
Non-manual occupation (Ref)			
Manual occupation		**1.75, 1.20**–**2.54**	**1.74, 1.19**–**2.53**
Work sector	–		
Other (Ref)			
Health & social care		**4.26, 2.63**–**6.90**	**4.18, 2.56**–**6.81**
Education & childcare		**2.35, 1.40**–**3.94**	**2.45, 1.45**–**4.14**
Key public service		**3.50, 1.70**–**7.20**	**3.78, 1.83**–**7.81**
Local & national govt		1.06, 0.51–2.22	1.07, 0.51–2.23
Food & essential goods		**2.56, 1.20**–**5.48**	**2.51, 1.17**–**5.37**
Public safety & security		**3.60, 1.29**–**10.1**	**3.90, 1.39**–**11.0**
Transport		**2.60, 1.18**–**5.72**	**2.60, 1.17**–**5.77**
Utilities, comms & finance		1.48, 0.89–2.45	1.45, 0.87–2.42
Index of multiple deprivation	–		
1st quartile (Ref)			
2nd quartile		0.97, 0.61–1.54	0.98, 0.62–1.56
3rd quartile		0.98, 0.62–1.56	0.99, 0.63–1.58
4th quartile		1.02, 0.63–1.65	1.10, 0.62–1.64
COVID-19–related financial hardship (15-point score)	–	**1.13, 1.07**–**1.20**	**1.14, 1.08**–**1.21**
Chronic illness of self	–		
No chronic illness (Ref)			
Chronic illness		0.61, 0.37–1.02	0.62, 0.37–1.02
Chronic illness of household member	–		
No chronic illness (Ref)			
Chronic illness		1.15, 0.70–1.86	1.14, 0.70–1.86
Vaccination status			
Not been vaccinated (Ref)			
Been vaccinated		**2.01, 1.29**–**3.11**	**2.08, 1.33**–**3.24**
**Block 3: Psychological variables**			
Worry about COVID-19 (5-point scale)	–	–	1.08, 0.91–1.28
Risk of COVID-19 to self (5-point scale)	–	–	1.12, 0.94–1.32
Believe had COVID-19	–		
Not had COVID-19 (Ref)			
Had COVID-19			0.66, 0.44–1.00
Believe Govt info is biased (5-point scale)	–	–	0.96, 0.84–1.10
Willing to leave home if with symptoms	–	–	
Not willing (Ref)			
Willing			1.05, 0.75–1.48

CI, confidence interval. Bolding denotes a significant association (*P* < .05 2-tailed).

a Weighted sample size for the analysis was 1194.

b Age was included as a quantitative variable, so the parameter is the odds ratio per additional year of age.

## Discussion

A substantial percentage of the UK population were attending their workplace in early 2021 even though they reported being able to work fully from home, contrary to UK Government guidance.^28^ Our estimate (27%) is similar to that reported by another survey also conducted in January to February 2021, which found that 31% of working adults were working on business premises.^29^ This suggests that there may be scope for reducing transmission by reducing the prevalence of this behaviour. Having been vaccinated, financial hardship, socio-economic grade C2DE, having a dependent child at home and working in certain key sectors were associated with higher likelihood of workplace attendance. Women were less likely to attend the workplace than men. These findings showing that different sociodemographic groups have been affected very differently by the pandemic, even within occupational groups such as scientists, are consistent with findings beyond the UK.^30^^,^^31^ Several of the predictors of workplace attendance could reflect targets for interventions aimed at reducing COVID-19 transmission.

The association between financial hardship and workplace attendance could mean that people who are struggling to meet their living costs feel greater pressure to attend than others. This may reflect a more precarious working environment, and there is evidence of employer pressure playing a role. The TUC has said that people who could work from home should not be pressured to attend workplaces, nor should they be given the option of doing so voluntarily.^10^ Increasing job security and reducing employer pressure could be addressed by government regulation. Greater financial and practical support for those asked to isolate could reduce those going out to work: findings from the CORSAIR study show that more than half of those even with symptoms are not isolating for the full period, and going out to work is one of the reasons given.^22^ In terms of the association with the presence of a dependent child in the household, it is notable that this exists even after adjusting for multiple financial variables within the data set.^23^ We note that the mental health of parents was affected by the first lockdown in the UK,^32^ which is consistent with other findings.^33^ One explanation may be that some parents attend work partly in order to reduce distress within the household. Another may be that although they can work at home when children are at school, they cannot do so easily when children are at home.

In April 2020, believing one had already had COVID-19 was shown to be associated with perceptions of immunity against the virus and reduced adherence to several protective behaviours in one UK sample.^34^ Although we did not observe an association between perceptions of prior infection and attending work, our findings of a positive association between reports of having been vaccinated and workplace attendance suggests that perceptions of immunity arising from the vaccine are now playing a similar role. Although the rollout of the vaccination programme in the UK has been rapid and is widely considered a success, there have been reported gaps in the provision of verbal and accessible written information explaining that immunity would take three weeks to build up, would be partial and it was possible that people could continue to be infectious, especially before the second dose. The absence of this information may be associated with a recent increase in self-reported breaches of current lockdown restrictions amongst older adults who were among the first to be vaccinated, where 41% of those older than 80 years reported having met someone outside of their household and support bubble less than 3 weeks after vaccination.^35^ A month or so into the programme, NHS England has provided scripts, posters and an animation for use alongside the vaccination programme.^36^ Hopefully, this will go some way to reducing the increased risky behaviours that can follow vaccination.

Increased workplace attendance in certain sectors suggests that these sectors may be considered for targeted interventions. In the case of health and social care, education and childcare and some other sectors, it could be that the fact that many front-line workers in these sectors need to attend the workplace leading to a culture in which other workers feel compelled to do so even if this is not necessary, something that may also have wider implications for attendance while sick.^33^ Personal communication suggests that another key reason for health and social care employees who could work at home not to do so is the lack of adequate digital technology for their work. This merits further examination.

In the UK, the Government's roadmap out of our third lockdown specifies a sequence of changes, starting with the reopening of schools and progressing to the removal of all legal limits on social contact. The need to proceed slowly through these changes has been emphasised repeatedly. Ensuring that a large number of people who can work from home do so is one key measure that can be taken to keep control of the pandemic as restrictions are eased. Given the importance of schools remaining open and the predicted increased transmission from children being at school,^37^ it is imperative that other measures are taken to keep COVID-19 under control. Providing a large number of people with the means to work at home when this is possible is one such measure, especially because this would reduce people interacting both in workplaces and on transport.

The limitations of our study include the following: 1) it relies on self-report which may cause reporting bias, particularly concerning whether work can be completed entirely from home – it is possible that although our respondents reported that they believed they could work from home, we do not know the circumstances of their employment to know whether this reflects the requirements of their role, 2) use of an online sample which, even though has been weighted to match major demographic features of the UK population, may nevertheless not be fully representative, 3) collinearity among predictors and 4) possible omission of other relevant variables. In relation to the latter point, research on broader contextual factors such as values and political orientations would provide another layer of understanding for the current findings.^38^^,^^39^

### Conclusions

Non-essential workplace attendance in the UK in early 2021 during the COVID-19 pandemic was substantial and significantly independently associated with a wide range of sociodemographic variables and personal circumstances. Having been vaccinated, financial hardship, manual occupational group, having a dependent child at home and working in certain key sectors were associated with higher likelihood of workplace attendance. These findings could inform government, employer-led and other interventions aimed at reducing non-essential workplace attendance in the future.

## Author statements

### Ethical approval

This work was conducted as part of service evaluation of the marketing and communications run by the Department of Health and Social Care and so did not require ethical approval.

### Funding

This work was funded by the 10.13039/501100002001National Institute for Health Research Health Services and Delivery Research Programme. LS, RA and GJR are supported by the National Institute for Health Research Health Protection Research Unit (NIHR HPRU) in Emergency Preparedness and Response, a partnership between 10.13039/501100002141Public Health England (PHE), King's College London and the University of East Anglia. RA is also supported by the NIHR HPRU in Behavioural Science and Evaluation, a partnership between PHE and the University of Bristol. HWWP receives funding from PHE and NHS England. NTF is partly funded by a grant from the UK Ministry of Defence. The views expressed are those of the authors and not necessarily those of the NIHR, PHE, the Department of Health and Social Care or the Ministry of Defence. Surveys were commissioned and funded by the Department of Health and Social Care (DHSC), with the authors providing advice on the question design and selection. DHSC had no role in analysis, decision to publish or preparation of the manuscript. Preliminary results were made available to the DHSC and the UK's Scientific Advisory Group for Emergencies.

### Data availability statement

The data are owned by the UK's Department of Health and Social Care, so no additional data are available from the authors.

### Competing interests

Authors received financial support from the UK's National Institute for Health Research for the submitted work. RW has undertaken research and consultancy for companies that manufacture smoking cessation medications (Pfizer and GSK). RA is an employee of Public Health England. HWWP receives additional salary support from Public Health England and NHS England. NTF is a participant of an independent group advising NHS Digital on the release of patient data. All authors are members of the UK's Scientific Advisory Group for Emergencies or its subgroups. There are no other financial relationships with any organisations that might have an interest in the submitted work in the previous three years and no other relationships or activities that could appear to have influenced the submitted work.
